# Morphohistometric evaluation of the embryological development of the heart in chicks

**DOI:** 10.2478/jvetres-2026-0042

**Published:** 2026-07-27

**Authors:** Muhammet Lütfi Selçuk, Fatma Kayıkcı Hekim

**Affiliations:** 1 Department of Physiotherapy and Rehabilitation, Department of Nutrition and Dietetics, Karamanaoğlu Mehmetbey University Faculty of Health Sciences, 70200 Karaman, Türkiye; 2 Department of Nutrition and Dietetics, Karamanaoğlu Mehmetbey University Faculty of Health Sciences, 70200 Karaman, Türkiye

**Keywords:** Cavalieri method, chick embryo, developmental embryology, heart

## Abstract

**Introduction:**

The aim of this study was to investigate the morphohistometric development of the chick heart at certain incubation periods using quantitative methods.

**Material and Methods:**

In the study, the hearts of 24 Babcock White Leghorn chicks, six each from the 10^th^, 13^th^, 16^th^ and 21^st^ days of incubation, were used. The hearts were weighed, and their diameters were measured with a digital caliper. Paraffin blocks of heart tissue were then prepared, and serial sections were taken at 5 μm and 10 μm thickness, stained with Crossmon’s triple staining method and photographed under a microscope. The areas of the sections were calculated in an image analysis tool, and the heart volume was calculated using the Cavalieri method.

**Results:**

It was observed that the right atrium wall was thicker than the left atrium wall on the 10^th^ and 13^th^ days, and that the left atrium wall became thicker than the right one on the16^th^ day still was on the 21^st^ day. The left ventricular wall was thicker than the right one. It was observed that the diameter, wall diameter and lumen area of the aorta increased significantly towards the last day of incubation. The lumen diameter of the aorta increased until the 16^th^ day but had decreased by the 21^st^ day.

**Conclusion:**

Besides contributing to existing anatomical knowledge with the presented morphometric data, these findings are expected to form a benchmark for imaging system data used in clinical practice, serve as reference values for clinical sciences and aid physicians in disease diagnosis.

## Introduction

The heart, located at the center of the cardiovascular system, consists of four chambers (5). While the atria receive blood flow draining from the venous system, the ventricles pump blood into the arterial system. The heart wall consists of three layers: the endocardium (innermost layer), the myocardium (middle layer) and the epicardium (outermost layer). The endocardium lines the lumen of the heart. This layer consists of endothelium (simple cuboidal epithelium), subendothelial connective tissue and subendocardium. The subendocardium is in contact with the heart muscle and contains small coronary blood vessels, nerves and Purkinje fibres in specific areas (39). The endocardium serves as a smooth lining for the heart’s four chambers and a covering for the atrioventricular valves (10). The myocardium is the thickest layer of the heart wall and makes up most of the heart. It consists of a branching columnar arrangement of cardiac muscle cells the ends of which are connected to each other by intercalated discs. This muscle contracts to pump blood out of the heart’s ventricles, distributing it to the body’s tissues and organs. The left ventricular myocardial wall is thicker than the right one because it must pump blood very far and overcome the high pressure and resistance of the systemic circulation. The walls of the atria are thinner than the walls of the ventricles. The myocardium of the right atrium is the thinnest because of the relatively low pressure and resistance of the blood flow. The muscle contractions of heartbeats are generated and regulated by the cardiac conduction system, including the sinoatrial node, atrioventricular node, atrioventricular bundle and Purkinje fibres (12, 18). The epicardium surrounds the heart. It is a layer of connective tissue containing nerves, blood vessels and fat cells. The inner surface of the epicardium is connected to the heart muscle, and its outer surface is covered with mesothelium facing the pericardial cavity. The mesothelium secretes a fluid known as pericardial fluid, which lubricates the space between the epicardium (visceral pericardium) and the parietal pericardium during the movements caused by heart contractions, reducing friction. The epicardium covers and protects the heart and provides nutrients and innervation as small blood vessels and nerves pass through it (10, 37).

The formation of the human heart is a studied in various models, but because the development of a bird’s heart is similar to that of a human heart, bird models are invaluable for research into the organ’s development. Like mammals, chick’s embryos develop within an amnion and a chorion, and developmental processes are highly conserved between amniotic fluid layers (14, 20). Bird embryos are considered an ideal model for studying the process from heart tube formation to the development of the four-chambered heart, as well as for examining congenital heart malformations (28, 32). Heart defects found in humans can be summarised in bird embryos (6). Similar to humans, bird embryos remain relatively flat from the early to the late gastrulation stages. This developmental similarity enables accelerated observation of both dorsal and ventral tissues through mounted *ex ovo* culture techniques. The complete nest *ex ovo* culture technique for avian embryos allows the study of cellular behaviour underlying heart and blood vessel morphogenesis under physiological conditions (3, 33). A morphological staging description scheme is used in such research: the Hamburger and Hamilton (HH) bird staging system is the standard and is a reliable method. To correlate embryonic development with heart development, the HH scale is used to describe the stages of cardiac development (17, 26).

Besides chicken eggs’ advantage to researchers of allowing easy planning of experiments (2, 39), their larger size also recommends them; *in ovo* manipulations, including tissue grafting, ablation or injection, can be performed through a small hole made in the egg chamber (5) and are rendered easier by the absence of a placental barrier in chick eggs. The facility of the creation of transgenic lines and live cell imaging, the direct adenoviral administration of genome engineering tools into the chick blastoderm, the creation of chimeras producing carriers of the targeted mutation; alternatively, the ability to transfect primitive germ cells and use them for germline transmission, increase their value as experimental animal (11, 21).

A literature search revealed no studies that histologically and morphologically examined the embryonic development of the heart in healthy chicks, including inspections of anatomical structures such as the heart diameter, atrium, ventricle and septa. In this study, the embryonic development of the heart was measured morphohistometrically using quantitative methods for diameter and thickness measurements, and the heart volume was calculated using stereological methods.

## Material and Methods

### Material

Twenty-four euthanised cadaver Babcock White Leghorn chicks, six each from the 10^th^, 13^th^, 16^th^ and 21^st^ days of incubation according to the HH scale (17), were used in this study. The research was approved by The Karamanaoğlu Mehmetbey University Faculty of Health Sciences Research Ethics Committee (2022/03-21).

### Sample collection

The weights of embryos euthanised on the 10^th^, 13^th^, 16^th^ and 21^st^ days of incubation were measured using a precision scale. The heart was removed from chick embryos fixed in 10% neutral buffered formalin (pH 7.4) *via* an abdominal incision, separating the heart from the surrounding organs, and the surrounding tissues were removed. The heart weight was weighed using a precision scale (Table 1). Relative organ weight was obtained by dividing the heart weight by the embryo weight.

**Table 1.** Chick embryo weight parameters and heart volumes at different periods (Mean±SD)

Parameter	Incubation day 10	Incubation day 13	Incubation day 16	Incubation day 21
Embryo weight (g)	3.11 ± 0.21^a^	8.91 ± 0.46^b^	22.74 ± 2.72^c^	41.18 ± 2.26^d^
Heart weight (g)	0.021 ± 0.002^a^	0.071 ± 0.007^b^	0.182 ± 0.031^c^	0.237 ± 0.062^d^
Relative organ weight (g)	0.686 ± 0.038^a^	0.793 ± 0.058^b^	0.804 ± 0.152^b^	0.577 ± 0.151^a^
Heart volume (mm^3^)	0.371 ± 0.05^a^	1.279 ± 0.11^b^	1.922 ± 0.43^c^	3.239 ± 0.57^d^

a, b, c and d –different superscript letters in the same row represent statistically significant differences (p < 0.001)

### Morphometric and histological analysis of the heart

The cranio-caudal, dorso-ventral and medio-lateral diameters of the heart were measured using a digital caliper (Table 2). After the measurements, the hearts were placed in tissue-tracking cassettes, washed in running water overnight, dehydrated in a graded alcohol series and cleared in xylene, and paraffin blocks were prepared. Using a rotating microtome, sagittal serial sections of 5 μm thickness were taken at regular intervals from each block for histological examinations and 10 μm thickness sections were taken every 20 sections for volume calculation. The sections were oven-dried at 37°C for 24 h and were stained with Crossmon’s trichrome stain. The sections were photographed and examined with a light microscope (Leica DM-2500 connected to a DFC-320 digital camera, Leica Microsystems, Wetzlar, Germany) (9, 35). The ImageJ Analysis Program was used for heart measurements (34). The following measurements of the heart were made:

**Table 2.** Chick embryo heart diameters at different periods (Mean±SD)

Diameter	Incubation day 10	Incubation day 13	Incubation day 16	Incubation day 21
Cranio-caudal (mm)	4.595 ± 0.411^a^	7.158 ± 0.369^b^	9.375 ± 0.768^c^	10.700 ± 0.769^d^
Dorso-ventral (mm)	2.662 ± 0.300^a^	3.753 ± 0.209^b^	5.607 ± 0.539^c^	6.007 ± 0.479^c^
Medio-lateral (mm)	3.507 ± 0.167^a^	4.658 ± 0.291^b^	6.510 ± 0.268^c^	7.513 ± 0.785^d^

a, b, c and d –different superscript letters in the same row represent statistically significant differences (p < 0.001)

- atrial wall thicknesses were measured at five different points at 10× magnification and averaged (Fig. 1). The left/right atrium ratio was calculated by dividing the left atrial wall thickness (LAW) by the right atrial wall thickness (RAW);

**Fig. 1.**
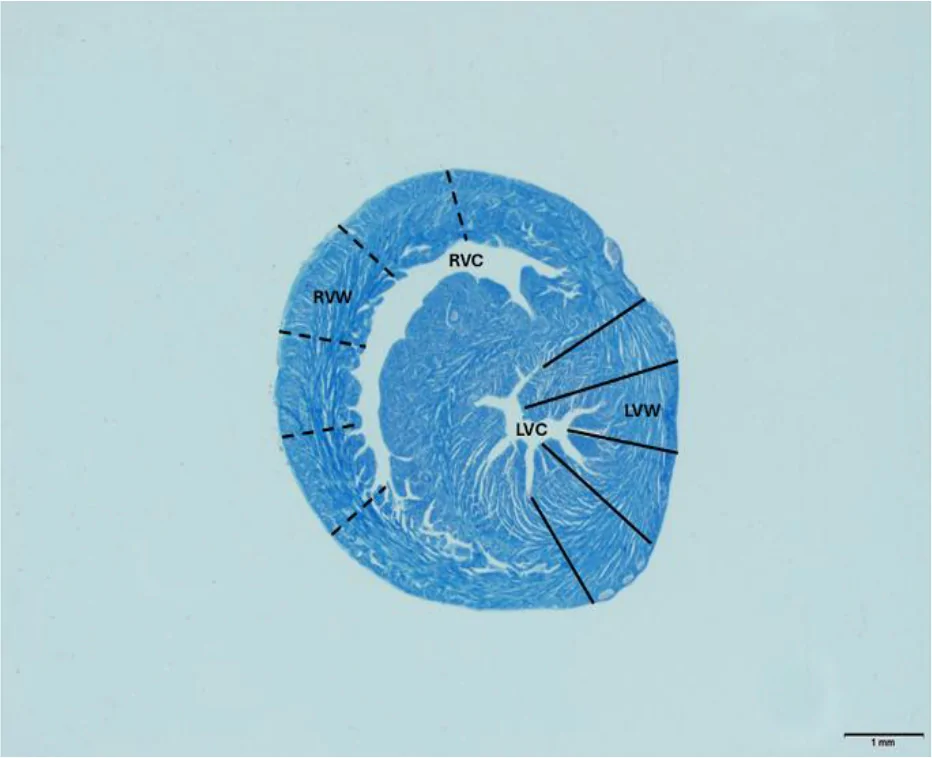
Morphometric measurement of the ventricular wall of heart. RVW – right ventricular wall; RVC – right ventricular cavity; LVW – left ventricular wall; LVC – left ventricular cavity. Crossmon’s trichrome staining. Scale bar – 1 mm

- ventricular wall thicknesses were measured at five different points at 10× magnification, and averages were taken. The left/right ventricular ratio was calculated by dividing the left ventricular wall thickness (LVW) by the right ventricular wall thickness (RVW);

- the thicknesses of the cardiac muscle layers (epicardium, myocardium and endocardium) forming the ventricles were measured at 40× magnification at five different points on the histological section. The average of the five measured values was calculated;

- the aortic wall thickness was measured symmetrically from four different points in each sample, and the average of the values was calculated (Fig. 2A);

**Fig. 2.**
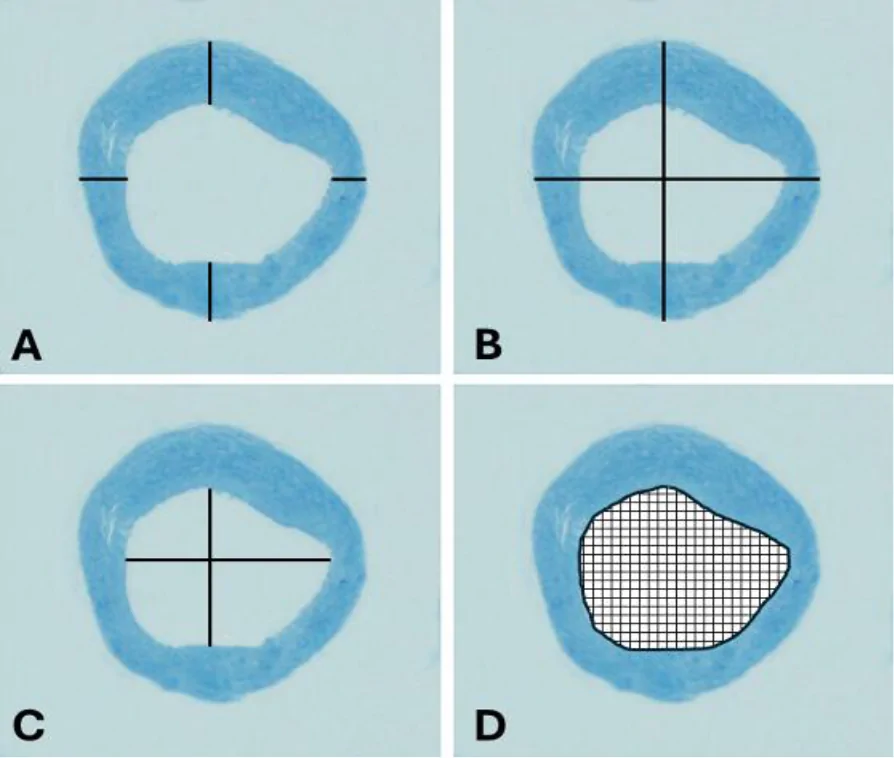
Morphometric measurement of sections of a chick embryo aorta. A – aortic wall thickness; B – aortic diameter; C – aortic lumen diameter; D – aortic lumen area

- the aortic diameter was calculated as the average of the largest and smallest outer aortic diameter values (Fig. 2B);

- the aortic lumen diameter was calculated as the average of the largest and smallest diameter values, taking the inner surface of the aortic wall as reference (Fig. 2C);

- the cross-sectional area of the aortic lumen (the inner surface of the aortic wall) was selected using the freehand selection feature of ImageJ Analysis. Based on this selection, the program automatically calculated its surface area (Fig. 2D).

### Volume calculation in the heart using Cavalieri’s method

The ImageJ program was calibrated, and a point counting grid (d = 0.1 mm) was placed on the cross-sectional images. A different marker was selected for each area of interest, and the points falling into the areas were counted separately (Fig. 3).

**Fig. 3.**
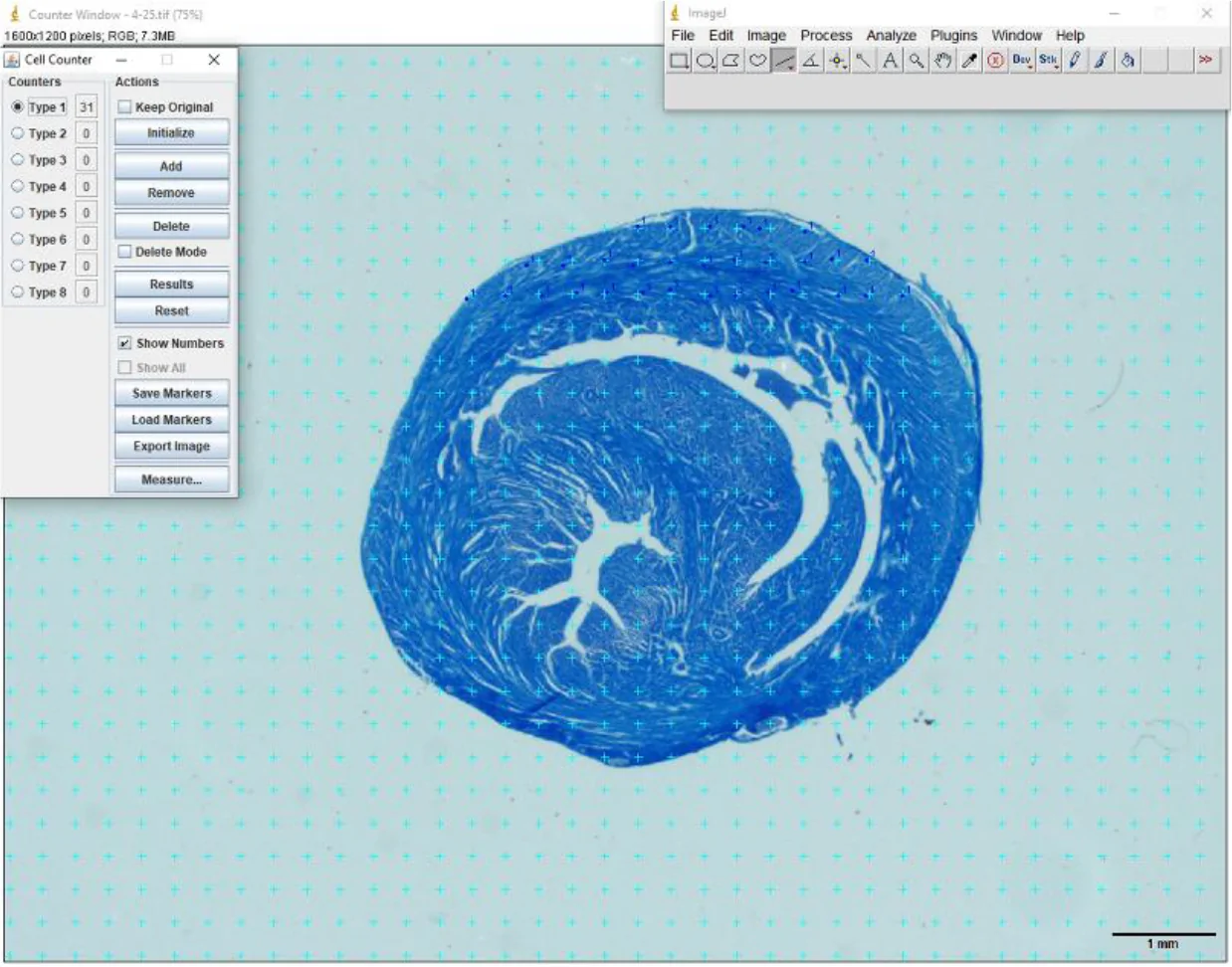
Application of the point counting grid for chick embryo heart sections

The volumes were estimated using the formula:

$$\text{V}=\left({\text{a}}_{\text{p}}\right)\times \sum \text{P}\times \text{t}$$

where V is the volume of the structure of the sample of interest, ap is the area of a point counting grid, ∑P is the total number of points falling on the structure of interest and t is the average section thickness (27, 36). The coefficient of error was calculated according to the relevant literature (16).

### Statistical analysis

Heart morphometric data were analysed using the SPSS v. 21.0 statistical package (IBM, Armonk, NY, USA). Normal distribution of variables was confirmed using the Kolmogorov– Smirnov and Shapiro–Wilk tests, as well as histograms and probability plots. One-way ANOVA was used for statistical comparisons of data obtained from groups. In cases where the P value indicated significant difference, pairwise post-hoc comparisons between statistically significant results were made using the Tukey test. Values were recorded as mean ± SD. A value of p < 0.05 was considered statistically significant.

## Results

### Morphometric evaluation of the heart

The average embryo weight, heart weight, relative organ weight and heart volume of chick embryos are presented in Table 1. The data indicate that embryo weight, heart weight and heart volume had increased significantly by the 21^st^ day (p < 0.001). The diameters of the embryos’ hearts are presented in Table 2. Analysis of the table reveals that diameter measurements increased towards the 21^st^ day of incubation.

While statistical differences were observed between the groups in the cranio-caudal and medio-lateral diameter measurements, no statistical difference was detected in the dorso-ventral heart diameter measurements taken on the 16^th^ and 21^st^ days.

The thickness measurements of the atriums, ventricles and septum of the embryos are presented in Table 3. When comparing the wall thicknesses of the atria, it was found that they increased towards the last day of incubation, but there was only a statistical difference in between the LAW on the 10^th^ day and the LAW on the 13^th^ day (p < 0.001). A comparison of the wall thickness between the left and right atria revealed that the right had significantly thicker walls on the 10^th^ and 13^th^ days, while the left had significantly thicker walls on the 16^th^ and 21^st^ days (p < 0.001). Although there was an increase in the left/right atrium ratio with longer days of incubation, it was not statistically significant (p > 0.05).

**Table 3.** Morphometric measurements of chick embryo heart walls at different periods (Mean±SD)

Parameter (thickness unless stated)	Incubation day 10	Incubation day 13	Incubation day 16	Incubation day 21
Left atrial wall (μm)	8.87 ± 0.88^a^	12.67 ± 2.08^ab^	15.75 ± 5.25^b^	16.40 ± 2.13^b^
Right atrial wall (μm)	11.47 ± 3.87	13.50 ± 2.80	13.45 ± 2.35	15.53 ± 4.97
Left/right atrium ratio	0.89 ± 0.44	0.95 ± 0.084	1.14 ± 0.18	1.16 ± 0.43
Left ventricular wall (μm)	60.53 ± 17.08^a^	98.56 ± 13.83^b^	115.22 ± 21.58^b^	156.28 ± 26.07^c^
Right ventricular wall (μm)	52.73 ± 6.32^a^	79.72 ± 12.68^a^	89.06 ± 30.19^a^	109.83 ± 30.41^b^
Left/right ventricle ratio	1.15 ± 0.31	1.22 ± 0.39	1.47 ± 0.35	1.48 ± 0.37
Interatrial septum (μm)	6.27 ± 1.79^a^	6.67 ± 1.25^a^	16.78 ± 6.78^b^	16.53 ± 5.09^b^
Interventricular septum (μm)	69.07 ± 8.13^a^	87.28 ± 20.13^a^	118.83 ± 15.52^b^	139.50 ± 15.74^b^

a, b and c –different letters in the same row represent statistically significant differences (p < 0.001)

Comparing ventricular wall thicknesses revealed that both the LVW and RVW increased towards the last day of incubation, and that there was a statistical difference between them (p < 0.001). The LVW was found to be significantly thicker than the RVW. An increase was noted in the left/right ventricle ratio over the incubation days; however, it did not reach statistical significance (p > 0.05).

When septum thicknesses were examined, it was determined that the thicknesses increased according to the incubation days, and that there was a statistical difference between the 10^th^ and 13^th^ days and the 16^th^ and 21^st^ days in both the interatrial septum and interventricular septum (p < 0.001).

The thicknesses of the epicardium, myocardium and endocardium layers forming the ventricular wall of embryo hearts are presented in Table 4. When the myocardium layers forming the ventricular wall were examined, it was determined that the right side layers of the heart were thicker than the left side layers, except for on the 16^th^ and 21^st^ days. This was statistically significant (p < 0.001).

**Table 4.** Chick embryo heart muscle layer thickness in the ventricular wall at different periods (Mean±SD)

Layer	Incubation day 10	Incubation day 13	Incubation day 16	Incubation day 21
Left epicardium (μm)	3.90 ± 0.33	5.49 ± 0.57	7.55 ± 1.16	14.24 ± 0.68
Right epicardium (μm)	5.01 ± 0.63	8.08 ± 1.46	8.42 ± 1.28	26.73 ± 2.48
Left myocardium (μm)	36.29 ± 5.14	52.26 ± 2.82	71.31 ± 7.13	73.05 ± 2.51
Right myocardium (μm)	38.78 ± 3.36	57.49 ± 3.18	62.53 ± 3.07	66.76 ± 2.71
Left endocardium (μm)	3.39 ± 0.24	4.73 ± 0.45	7.06 ± 0.97	7.48 ± 0.73
Right endocardium (μm)	4.29 ± 0.46	6.63 ± 0.15	7.40 ± 0.66	10.13 ± 0.56

Measurements of the aortic wall thickness and aortic lumen on each incubation day are given in Table 5. It was observed that aortic wall thickness and aortic diameter increased towards the last day of incubation, with a statistical difference between the 13^th^ and 16^th^ days (p < 0.001). The aortic lumen area increased as the last day of incubation approached, significantly differently on the 16^th^ and 21^st^ days. Aortic lumen diameter increased until the 16^th^ day of incubation and then decreased on the 21^st^ day, but the reduction had no statistical significance (p > 0.05).

**Table 5.** Morphometric measurements of the chick embryo aorta at different periods (Mean±SD)

Parameter	Incubation day 10	Incubation day 13	Incubation day 16	Incubation day 21
Aortic wall thickness (μm)	15.13 ± 1.90^a^	20.28 ± 2.27^b^	29.26 ± 4.22^c^	30.35 ± 3.12^c^
Aortic diameter (μm)	52.29 ± 5.34^a^	71.22 ± 4.31^b^	107.84 ± 10.83^c^	110.64 ± 11.71^c^
Aortic lumen area (μm^2^)	342.65 ± 14.12^a^	729.82 ± 26.53^b^	1864.95 ± 88.15^c^	1912.57 ± 75.34^c^
Aortic lumen diameter (μm)	21.23 ± 1.37^a^	31.54 ± 3.06^a^	77.88 ± 11.87^b^	70.35 ± 8.47^b^

a, b and c –different superscript letters in the same row represent statistically significant differences (p < 0.001)

Coefficient of error values for embryonic heart volumes are shown in Fig. 4. The average value was calculated as 0.009 ± 0.004.

**Fig. 4.**
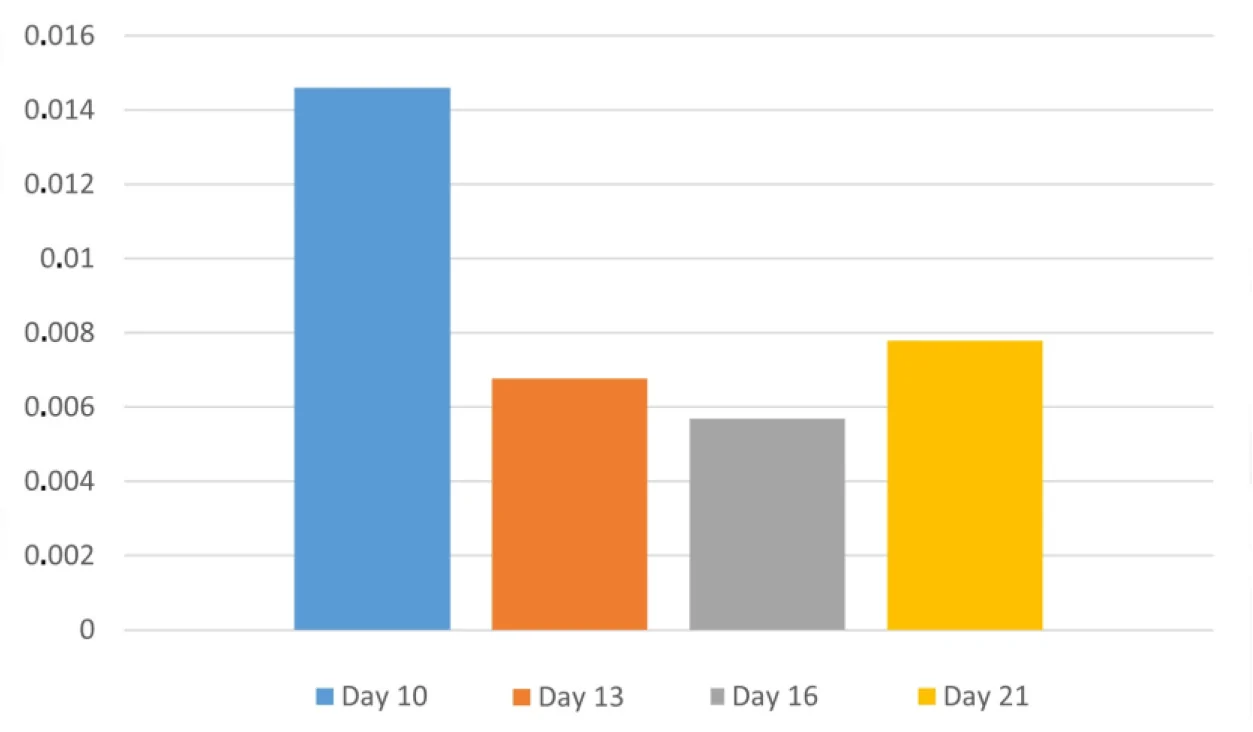
Coefficients of error in chick embryonic heart volumes

### Embryological development of the heart

On the 10^th^ embryonic day (HH stage 36), it was observed that the heart wall consisted of the three layers: endocardium, myocardium and epicardium. On this embryonic day, it was determined that the ventricular septation stage of the heart was complete, and primitive ventricles had formed. The atrial septation phase was observed to be ongoing, with vessels entering and exiting these chambers. The heart valves had formed and were in a structure that could pump blood from the heart. The right tricuspid valve was in the form of a muscle band (tricuspid band). The heart wall was rich in blood vessels, and coronary vasculature was seen extending from the subepicardial layer to the myocardium. On the 16^th^ day of incubation, coronary vascularisation was well developed. Vascular wall layers began to be seen from the 10^th^ embryonic day onwards, and these structures could be easily distinguished on the day of hatching. Purkinje fibres, which are part of the cardiac conduction system and formed as a result of the differentiation of cardiomyocytes, were frequently observed on the subendocardial surface of the right and left ventricles. Purkinje fibres were larger and had paler sarcoplasm than cardiomyocytes. The right ventricular lumen was filled with blood cells at all embryonic stages. On the 10^th^ embryonic day, although transverse banding, formation of intercalated discs, and collaterals were not clearly seen in the cardiomyocytes forming the myocardium, a primitive myocardium was discernible.

From the 13^th^ embryonic day of incubation (HH stage 39), the histological structure in cardiomyocytes began to take shape. All these histological findings continued to develop throughout the subsequent embryonic days. On the hatching day (HH stage 46), it was observed that the atrial septation and Purkinje fibre network of the heart were complete, and the heart was functionally a four-chambered organ. In this appearance, the embryonic chick heart resembled the heart structure of an adult chicken (Fig. 5).

**Fig. 5.**
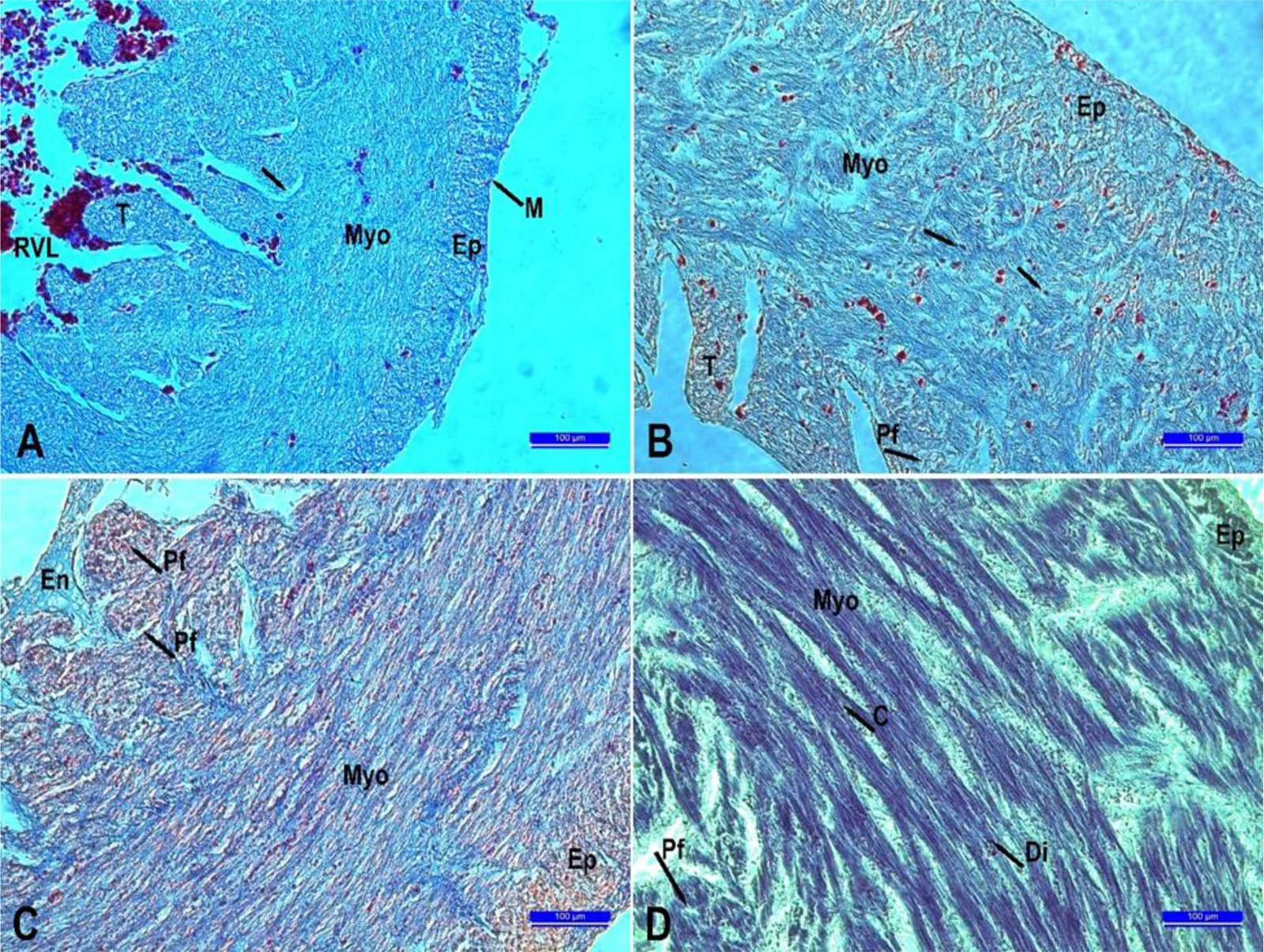
Histological development of the heart according to different periods. A. 10^th^ day. B. 13^th^ day. C.16^th^ day. D. 21^st^ day. C – Collateral; Di – Intercalary disc; En – Endocardium; Ep – Epicardium; M – Mesothelium; Myo – Myocardium; Pf – Purkinje fibers; T – Trabecula; RVL – right ventricular lumen; → – cardiomyocyte nucleus. Crossmon’s trichrome staining. Scale bars = 100 μm

## Discussion

The heart is the first organ to develop in vertebrates. Once the primordial structure of the heart forms, it continues to grow and change. The development, anatomy and physiology of the heart in chick embryos are quite similar to those in human foetuses. Because of this similarity, embryonic chicks are considered a reliable animal model for studying heart development and embryology (4).

Chick embryos are frequently preferred in experimental studies because their eggs are large and allow *in ovo* manipulations such as tissue grafting, ablation or injection to be performed easily (8, 39). They are valuable tools for understanding disease pathogenesis and progression in congenital heart diseases, as well as for investigating the underlying mechanisms. Additionally, this model can be used to test preclinical therapeutic interventions (23). In our research, Babcock White Leghorn chick hearts were evaluated on the 10^th^, 13^th^, 16^th^ and 21^st^ days of incubation. The diameters of the hearts, the heart muscle and the atrial and ventricular wall thicknesses were measured.

In the study, chick embryo weights, heart weight and heart volume showed a significant increase with longer days of incubation. Relative organ weights were observed to increase from the 10^th^ to the 16^th^ day of incubation but to decrease on the 21^st^ day. This decrease was attributed to the rapid growth of the heart between days 16 and 21. When the heart diameters were examined, a significant increase was observed towards the 21^st^ day of incubation, resulting from rapid growth.

In the examination of the heart wall thicknesses, it was observed that the wall thickness of both atria increased towards the last day of incubation. The RAW was thicker than the LAW on the 10^th^ and 13^th^ days, and the LAW had become thicker than the RAW on the 16^th^ day and remained so until the 21^st^. When the ventricular wall thicknesses were examined, it was seen that the LVW was thicker than the RVW. As organ development accelerates towards the last day of incubation, and more blood is needed, the right side of the heart increases its workload, resulting in differences in the thicknesses of the right and left walls. The thicknesses of the interatrial septum and interventricular septum increased towards the last day of incubation. In morphometric measurements of the aorta, the its wall diameter, overall diameter and lumen area increased significantly towards the last day of incubation. It was determined that the lumen diameter of the aorta increased until the 16^th^ day but had decreased by the 21^st^ day. This decrease was thought to be due to the pressure exerted by the growth of adjacent organ primaries and the expansion of the aortic lumen area.

In volume calculations made with the Cavalieri method, the error coefficient being 10% or less is an important parameter for the reliability of the research (16, 36). In the research conducted, this value was calculated separately for the heart and given in Fig. 4, and the stated values are reliable.

Research reports that cardiac progenitor cells begin to form the endocardium, myocardium and parietal pericardium, which are the wall layers of the heart, in the 12^th^–13^th^ h of incubation (HH scale, stage 3) (7, 26). Männer (24) stated that the epicardium was completely formed on the fifth day of incubation (HH stage 27). At 51–56 h of incubation (HH stage 16), the myocardium consisted of a compact layer and a trabecular layer adjacent to the epicardium (31). The first morphological change in the primitive ventricular wall during this incubation period is the formation of projections called trabeculae, which will contribute to ventricular septation in later stages. These trabeculae begin to grow and thicken. At approximately stage 34 on the HH scale, the myocardium develops a mature network of trabeculae (29). The increase in myocardial mass is due to trabeculations (19).

Cardiac myocyte proliferation, which is particularly high between the 8^th^ and 14^th^ days of incubation, continues until the egg hatches. However, cardiac myocyte proliferation during this period increases the size of the heart not only through hyperplasia, but also through hypertrophy, characterised by an increase in cell size (22). Atrial trabeculations were reported to appear at HH stage 27 of incubation (1). In this study, the findings in the embryonic heart from day 10 of incubation were consistent with those of previous investigators. During HH stage 16, the myocardium causes the endothelial cells in the endocardium to transform into mesenchymal cells. These mesenchymal cells contribute to the formation of the mitral and tricuspid valves and to atrial and ventricular septation (13, 26). Some researchers reported that the embryo at HH stage 34 has a four-chambered heart structure with valves (26). This four-chambered heart is the result of the complex process of cardiac septation (25). The development of the endocardial cushion, which forms at HH stage 12, plays an important role in the formation of atrial and ventricular septation in the embryonic heart.

It is reported that the atrial septum begins to form in HH stage 16, and the ventricular septum in stage 17 (13). No differently in birds, the atrial septum begins to form in the 16^th^ HH stage of incubation and completes its formation on the day of egg hatching (26, 30). It is reported that ventricular septation is complete by the 32^nd^ or 33 ^rd^ HH stage of incubation (38). In this study, it was determined that the right and left atria were developing during subsequent incubation periods, and that ventricular septation was completed by the 10^th^ embryonic day (HH stage 36), which are observations consistent with those in the literature. Purkinje fibres have been categorised as connexin-42 cells adjacent to blood vessels which continue to develop in HH stages 36–46 (15, 26). The development of valves and Purkinje fibres as seen in the present study was consistent with the cited researchers’ findings.

## Conclusion

Literature reviews revealed that morphometric studies on chick embryos are quite limited, and that quantitative methods and reference values for evaluating heart development are particularly restricted. Chick embryos share significant similarities in heart development, anatomy and physiology with human fetuses, making them invaluable for heart research. The data collected from this research, along with the identified relationships between the morphometric measurements, will greatly enhance our understanding of the region’s morphology. Additionally, the heart volume calculated using the Cavalieri method, combined with histological analysis, will provide valuable insights for future studies on the heart.
